# Supplementary material for: Modular assembly of proteins on nanoparticles
Source: Nat Commun. 2018 Apr 16;9:1489. doi: 10.1038/s41467-018-03931-4 (PMC5902510; doi:10.1038/s41467-018-03931-4)
Supplement: Supplementary file 1 — Supplementary Information [file 41467_2018_3931_MOESM1_ESM.pdf]

# Supplementary Information

## Modular assembly of proteins on nanoparticles

Ma *et al.*

Supplementary Note 1

**Protein-nanoparticle binding model**. To describe the binding of a protein (P) to a GNP surface, we assumed an equilibrium between P and free surface binding sites (S) as depicted in Supplementary Figure 1, where PS represents a surface site occupied by a protein P. The equilibrium is described by a dissociation constant $K_D$, under the assumption that only one molecule can bind to a single binding site. The dissociation constant can be calculated from the concentrations of the species at equilibrium according to the following:

$$P + S \rightleftharpoons PS;\ K_D = \frac{[P][S]}{[PS]} \qquad (1)$$

We define the total surface available $[S_0]$ as the sum of the free surface [S] and the occupied surface [PS]. By substituting [S] in the $K_D$ equation and rearranging we obtain the saturation one-site binding model below.

$$[PS] = [S_0]\frac{[P]}{K_D+[P]}; [S_0] = [S] + [PS] \qquad (2)$$

In a dynamic light scattering experiment, the concentration of the protein [P] is the independent variable, whereas the concentration of the bound protein [PS] is not directly measurable, but in this model it is proportional to the measured increase of diameter d of the nanoparticles (Δd). Similarly, $[S_0]$ is proportional to the maximum diameter increase ($\Delta d_{MAX}$) in such a way that the saturation binding equation becomes the below:

$$\Delta d = \Delta d_{MAX} \frac{[P]}{K_D + [P]}; [\mathrm{PS}]/[S_0] = \Delta d / \Delta d_{MAX} \qquad (3)$$

Δd can be calculated by subtracting the measured diameter (Z-average size) of naked nanoparticles to the measured diameter of the particles at each concentration [P]. By fitting Δd vs [P] data it is possible to estimate the values of the parameters $K_D$ and $\Delta d_{MAX}$.

It has to be pointed out that the concentration used in the experiment is not exactly coincident with [P], as a small proportion of the added molecule would bind to the surface and contribute to [PS]. Therefore, the model is valid only when the depletion of the experimental concentration is negligible. This is true when $[P] \gg [S_0]$, which applies in our experimental conditions. In fact, [P] was up to 1 µM, whereas an estimated $[S_0]$ would be reasonably below 50 nM, based on a concentration of 40 nm GNPs at OD 1 of ~150 pM and a maximum theoretical coverage of ~340 molecules per particle. This is the maximum number of spheres $N_0$ having a radius $R_H$ of ~2.4 nm (GST hydrodynamic radius) that can surround a sphere of diameter d = 40 nm, according to the empirical equation below:[1]

$$N_0 = 0.65 \frac{R_2^3 - R_1^3}{R_H^3};\ R_1 = {}^{d}/_{2};\ R_2 = R_1 + 2R_H \qquad (4)$$

Whereas $K_D$ can be conveniently used to indicate the binding affinity that a protein has towards the nanoparticle, $\Delta d_{MAX}$ has a less obvious meaning. Being proportional to the maximum number of binding sites available $S_0$, it can be used to compare the load of a given protein on the particle surface in different conditions. However, its absolute value shouldn't be confused with the maximum size of a nanoparticle covered by a complete corona, as the load, and therefore $\Delta d_{MAX}$, can vary depending on specific conditions.

## Supplementary Methods

**Detailed molecular dynamics simulation procedure**. The GROMOS G54a7 force field[2] was used for the interactions parameters in all the simulations. Gold atoms were considered neutral and the Lennard-Jones (LJ) parameters were taken from the literature. The atoms were position restrained to the crystal structure in all the simulations and interactions among the Au atoms were neglected. The model of the citrate molecule was parameterized by calculating the electronic structure and geometric properties using Density Functional Theory method with a BLYP3P functional and the 6-31G** basis set. The partial charges were calculated by fitting the electronic density using the ChelpG procedure (see Supplementary Table 3).[3] Gaussian03 package[4] was used for all the quantum mechanics calculations. The parameters for bonded interactions and LJ were adapted from the GROMOS54 A7 library (see Supplementary Table 3 for the

GROMOS atom types). The SPC water model[5] was used for water molecules. MD simulations were carried out at constant temperature and pressure. Initial velocities for each atom were generated with the Maxwell distribution at 293 K. The temperature was kept constant at 293 K using the V-rescale thermostat[6] with a coupling constant of 0.1 ps in all the simulations. The pressure was maintained constant at 1 bar using Berendsen barostat[7] with a coupling constant of 0.5 ps. The bond-lengths were constrained using LINCS[8] algorithm. An integration time step of 2 fs was used in all the simulations. The SETTLE algorithm was used to constrain bond lengths and bond angles of water molecules.[9] A dielectric permittivity, $\varepsilon_r$=1, and a time step of 2 fs were used in all the simulations. Electrostatic interactions were evaluated using particle mesh Ewald method[10] with a real space cut-off of 1.4 nm, grid spacing of 0.12 nm and a fourth order spline interpolation. Lennard-Jones interactions were truncated at 1.4 nm and the pair-list was updated every 5 time steps. The gold atoms were kept in their crystal position using position restraints with a force constant of 9000 kJ/nm$^2$. All the systems were energy minimized for at the least 1000 steps using the steepest descents method in order to remove any short distance clashes of the atoms in the generated solvent molecules. Subsequently, the density of the system was allow to adjust to the equilibrium value by performing 100 ps of molecular dynamics with position restraints only on the protein heavy atoms.

## Supplementary Figures

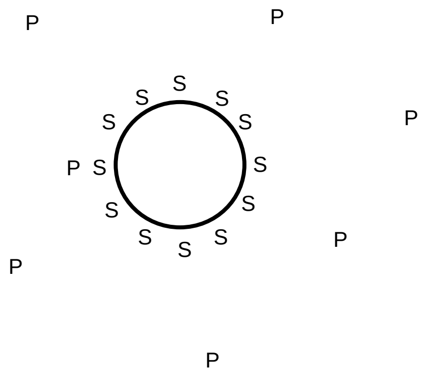


**Supplementary Figure 1**. Protein-nanoparticle binding model. Protein molecules (P) bind to a GNP surface at free surface binding sites (S). PS represents a surface site occupied by a protein P. Supplementary Equations 1-4 are valid under the assumption that only one molecule can bind to a single binding site.

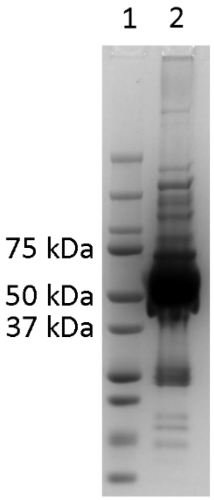


**Supplementary Figure 2**. SDS-PAGE of human serum used in the experiment of Figure 1C. The amount loaded here is representative of the same concentration used in the 3 hours incubation with nanoparticles. Lane 1: protein marker (relevant molecular weights are reported on the left); lane 2: human serum.

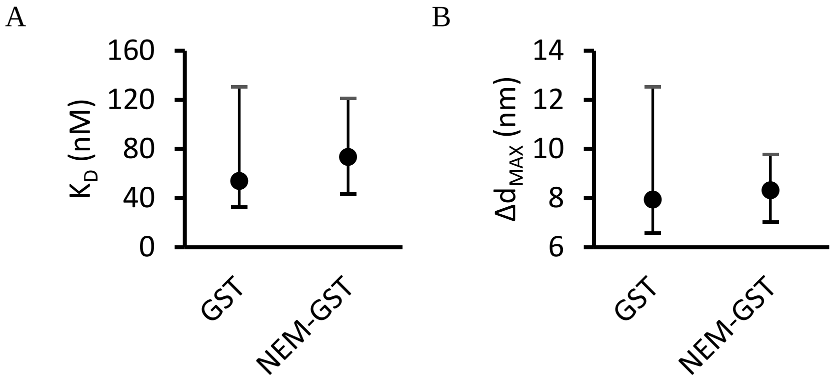


**Supplementary Figure 3**. 95% confidence intervals of $K_D$ (A) and $\Delta d_{MAX}$ (B) estimates for NEM-GST/GNPs compared to GST/GNPs. Data points represent the best estimates from the fit and the vertical bars represent the 95% confidence intervals.

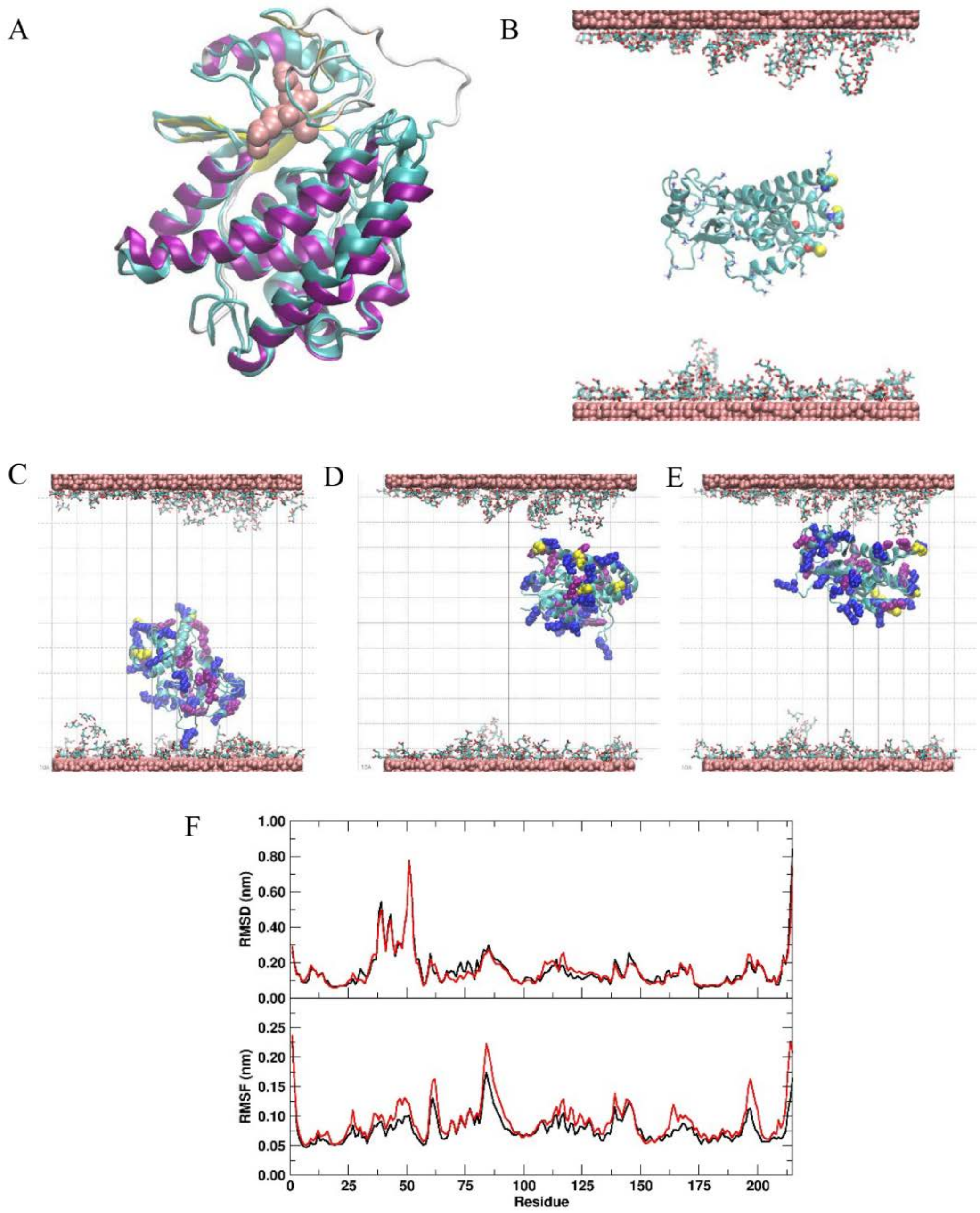


**Supplementary Figure 4**. Molecular dynamics of GST on GNPs. (A) Preparation of GST structure file for molecular dynamics (PDB ID: 1UA5 [http://dx.doi.org/10.2210/pdb1UA5/pdb]). GST after 100 ns MD simulation for equilibration purpose, compared to the crystallographic structure (cyan) after least-square fitting the backbone atoms. The C-terminal region comprising amino acids from the expression vector is visible on the top-right. The ligand glutathione from the crystal structure is also shown for reference and it is represented using Van-der-Waals sphere and colored in pink. (B) Starting conformation of GST on the

citrate coated gold surface. Positively charged residues and cysteine are showed in stick and van-der-Waals representation, respectively. (C-E) Conformations after 3 distinct 20 ns simulations. The one in panel C is the same conformation depicted in the main paper. The size of the grid corresponds to 10 Å. (F) Backbone RMSD of the protein after 20 ns simulation with respect to the initial structure (upper panel) and backbone root mean square fluctuations of the same in the simulation of panel C (bottom panel). Black line identifies the protein in solution, whereas red line is for the protein on gold surface. The structure of GST on GNPs does not present evident changes compared to the same in solution after 20 ns simulation.

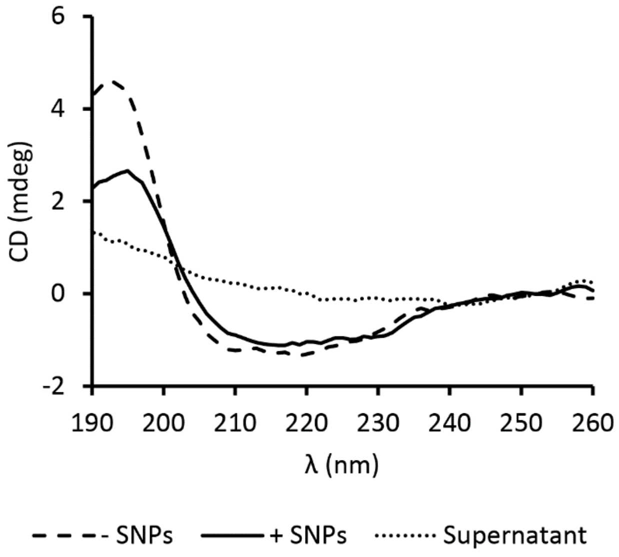


**Supplementary Figure 5**. SRCD spectra in the far UV region of GST in solution (dashed line) compared to GST bound to citrate capped 40nm SNPs (solid line) measured at the same GST concentration. The latter were incubated with excess GST for 1 hour and extensively washed before measurement to avoid the presence of protein in solution. After measurement SNPs were removed by centrifugation and a spectrum of the supernatant only (dotted line) was acquired and showed that little, if any, protein was present in solution, confirming that the spectrum of GST/SNPs was representative of the conformation of GST on silver surface.

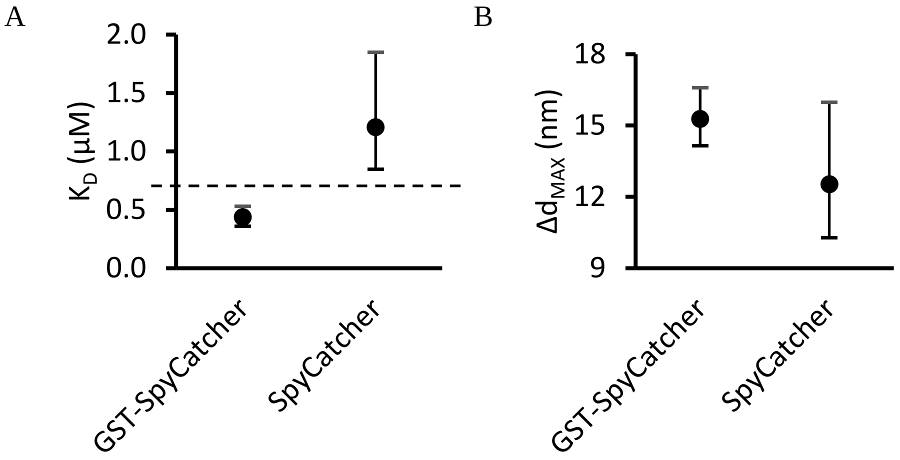


**Supplementary Figure 6**. 95% confidence intervals of $K_D$ (A) and $\Delta d_{MAX}$ (B) estimates for GST-SpyCatcher/GNPs compared to SpyCatcher/GNPs. Data points represent the best estimates from the fit and the vertical bars represent the 95% confidence intervals. The dashed horizontal line highlights the significance of the difference between the two estimates of $K_D$.

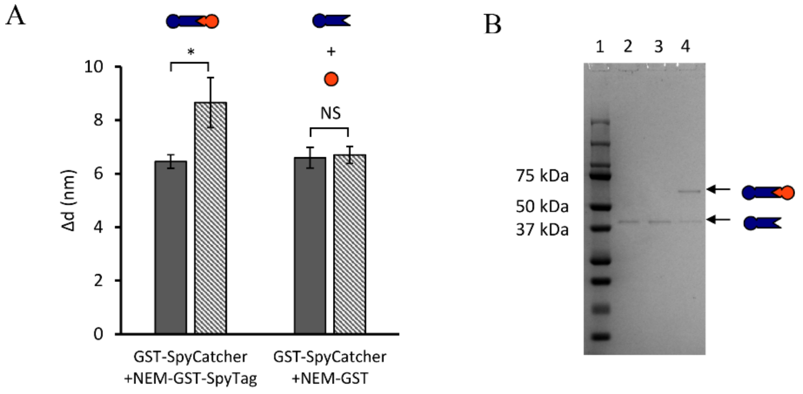


**Supplementary Figure 7**. Hierarchical assembly of GST-SpyCatcher and SpyTag on silver nanoparticles (SNPs). (A) Δd of GST-SpyCatcher/SNPs before (dark grey) and after (light grey) addition of either NEM-GST-SpyTag (left) or untagged NEM-GST (right). Tagging NEM-GST with SpyTag results into a significant increase of Δd, suggesting that a second layer of protein extensively binds to GNPs. Δd are averaged over three measurements with error bars representing standard deviation. t test results with a 95% confidence interval are indicated by * (significant, p=0.0167) and NS (non-significant, p= 0.7307). (B)

SDS-PAGE of proteins bound to SNPs. Lane 1: protein marker (relevant molecular weights are reported on the left); lane 2: GST-SpyCatcher/SNPs before any further incubation; lane 3: GST-SpyCatcher/SNPs incubated with untagged NEM-GST; lane 4: GST-SpyCatcher/SNPs incubated with NEM-GST-SpyTag. A band corresponding to GST-SpyCatcher-NEM-GST-SpyTag appears in lane 4, suggesting formation of the isopeptide bond between the two layers of protein corona.

## Supplementary Tables

| **Protein** | **MW (kDa)** | **Sequence** | **ENA accession number** |
|---|---|---|---|
| GST | 28.6 | mspilgywkikglvqptrlllleyleekyeehlyerdegdkwrnkkfelglefp<br>nlpyyidgdvkltqsmaiiryiadkhnmlggcpkeraeismlegavldirygv<br>sriayskdfetlkvdflsklpemlkmfedrlchktylngdhvthpdfmlydal<br>dvvlymdpmcldafpklvcfkkrieaipqidkylksskyiawplqgwqatfgg<br>gdhppksd<u>lvprgs</u>pgisgggggildsmgrlelklnss | LT986714 |
| GST-SpyCatcher | 38.5 | mspilgywkikglvqptrlllleyleekyeehlyerdegdkwrnkkfelglefp<br>nlpyyidgdvkltqsmaiiryiadkhnmlggcpkeraeismlegavldirygv<br>sriayskdfetlkvdflsklpemlkmfedrlchktylngdhvthpdfmlydal<br>dvvlymdpmcldafpklvcfkkrieaipqidkylksskyiawplqgwqatfgg<br>gdhppksd<u>lvprgs</u>GAMVDTLSGLSSEQGQSGDMTIEEDSATHIKFSKRDEDG<br>KELAGATMELRDSSGKTISTWISDGQVKDFYLYPGKYTFVETAAPDGYEVATA<br>ITFTVNEQGQVTVNGKATKGDgs | LT986711 |
| GST-SpyTag | 27.9 | mspilgywkikglvqptrlllleyleekyeehlyerdegdkwrnkkfelglefp<br>nlpyyidgdvkltqsmaiiryiadkhnmlggcpkeraeismlegavldirygv<br>sriayskdfetlkvdflsklpemlkmfedrlchktylngdhvthpdfmlydal<br>dvvlymdpmcldafpklvcfkkrieaipqidkylksskyiawplqgwqatfgg<br>gdhppksd<u>lvprgs</u>AHIVMVDAYKPTKgs | LT986708 |

**Supplementary Table 1**. Color-coded amino acid sequences of all recombinant proteins used. The molecular weight (MW) is indicated for the full protein, before any proteolytic cleavage. The underlined amino acid pattern <u>lvprgs</u> represent a thrombin cleavage site. This was used to obtain SpyCatcher from GST-SpyCatcher (thrombin cleaves between r and g residues). Capital letters represent residues from the target sequence introduced by cloning, whereas small letters are due to expression vector elements. Cysteine residues that can potentially bind GNPs or N-ethilmaleimide are highlighted in light blue, whereas the isopeptide bond-forming lysine (K) and aspartate (D) residues on SpyCatcher and SpyTag respectively are highlighted in red. The European Nucleotide Archive (ENA) accession number of the coding DNA sequence used for each protein expression is also indicated.

| System | Protein | Water | Na+ | Citrate | Gold atoms |
|---|---|---|---|---|---|
| **GST** | 1 | 22073 | 3 | 0 | 0 |
| **Au layers** | 0 | 33672 | 768 | 256 | 10000 |
| **Au+GST** | 1 | 32204 | 624 | 207 | 10000 |

**Supplementary Table 2.** Composition of the MD simulated systems.

| # | Atom type | Charge group | Partial Charges |
|---|---|---|---|
| 1 | O | 1 | -0.635 |
| 2 | C | 1 | 0.270 |
| 3 | O | 1 | -0.635 |
| 4 | CH2 | 2 | -0.300 |
| 5 | CH1 | 2 | 0.800 |
| 6 | C | 2 | 0.270 |
| 7 | O | 2 | -0.635 |
| 8 | O | 2 | -0.635 |
| 9 | OA | 3 | -0.600 |
| 10 | H | 3 | 0.400 |
| 11 | CH2 | 4 | -0.300 |
| 12 | C | 4 | 0.270 |
| 13 | O | 4 | -0.635 |
| 14 | O | 4 | -0.635 |

```
         O (1)
         |
    (2)  C — O (3)
         | (4)
         CH2
   (6)   |(5) (9)
O — C— CH1— OA -H (10)
(7)  |   |
     O   CH2 (11)
   (8)   |
   (12)  C — O (13)
         |
         O (14)
```

**Supplementary Table 3.** GROMOS atom types and partial charges for the citrate model.

## Supplementary References


1. Laera, S. *et al.* Measuring protein structure and stability of protein-nanoparticle systems with synchrotron radiation circular dichroism. *Nano Lett.* **11,** 4480–4 (2011).

2. Schmid, N. *et al.* Definition and testing of the GROMOS force-field versions 54A7 and 54B7. *Eur. Biophys. J.* **40,** 843–856 (2011).

3. Breneman, C. M. & Wiberg, K. B. Determining atom-centered monopoles from molecular electrostatic potentials. The need for high sampling density in formamide conformational analysis. *J. Comput. Chem.* **11,** 361–373 (1990).

4. Frisch, A. Gaussian 03. User's reference: manual version 7.1 (corresponding to Gaussian 03 revision D. 1). (2005).

5. Berendsen, H. J. C., Postma, J. P. M., van Gunsteren, W. F. & Hermans, J. Interaction models for water in relation to protein hydration. *Intermol. Forces* 331–342 (1981).

6. Bussi, G., Donadio, D. & Parrinello, M. Canonical sampling through velocity rescaling. *J. Chem. Phys.* **126,** 14101–14107 (2007).

7. Berendsen, H. J. C., Postma, J. P. M., Vangunsteren, W. F., Dinola, A. & Haak, J. R. Molecular-Dynamics with coupling to an external bath. *J. Chem. Phys.* **81,** 3684–3690 (1984).

8. Hess, B., Bekker, H., Berendsen, H. J. C. & Fraaije, J. G. E. M. LINCS: A linear constraint solver for molecular simulations. *J. Comput. Chem.* **18,** 1463–1472 (1997).

9. Miyamoto, S. & Kollman, P. Settle: An analytical version of the SHAKE and RATTLE algorithm for rigid water models. *J. Comput. Chem.* **13,** 952–962 (1992).

10. Darden, T., York, D. & Pedersen, L. Particle Mesh Ewald - an N.Log(N) method for Ewald sums in large systems. *J. Chem. Phys.* **98,** 10089–10092 (1993).
